# Comparative Cardioprotective Effectiveness: NOACs vs. Nattokinase—Bridging Basic Research to Clinical Findings

**DOI:** 10.3390/biom14080956

**Published:** 2024-08-07

**Authors:** Maja Muric, Marina Nikolic, Andreja Todorovic, Vladimir Jakovljevic, Ksenija Vucicevic

**Affiliations:** 1 Department of Physiology, Faculty of Medical Sciences, University of Kragujevac, 34000 Kragujevac, Serbia; majanikolickg90@gmail.com (M.M.); vladimir.jakovljevic@fmn.kg.ac.rs (V.J.); 2 Center of Excellence for Redox Balance Research in Cardiovascular and Metabolic Disorders, 34000 Kragujevac, Serbia; ksenija.vucicevic.kg@gmail.com; 3 Department of Cardiology, General Hospital Ćuprija, 35230 Ćuprija, Serbia; andrejatodorovic@gmail.com; 4 Department of Human Pathology, First Moscow State Medical, University IM Sechenov, 119991 Moscow, Russia; 5 Department of Pharmacy, Faculty of Medical Sciences, University of Kragujevac, 34000 Kragujevac, Serbia

**Keywords:** NOACs, nattokinase, cardioprotection, molecular mechanisms

## Abstract

The use of non-vitamin K antagonist oral anticoagulants (NOACs) has brought a significant progress in the management of cardiovascular diseases, considered clinically superior to vitamin K antagonists (VKAs) particularly in the prevention and treatment of thromboembolic events. In addition, numerous advantages such as fixed dosing, lack of laboratory monitoring, and fewer food and drug-to-drug interactions make the use of NOACs superior to VKAs. While NOACs are synthetic drugs prescribed for specific conditions, nattokinase (NK) is a natural enzyme derived from food that has potential health benefits. Various experimental and clinical studies reported the positive effects of NK on the circulatory system, including the thinning of blood and the dissolution of blood clots. This enzyme showed not only fibrinolytic activity due to its ability to degrade fibrin, but also an affinity as a substrate for plasmin. Recent studies have shown that NK has additional cardioprotective effects, such as antihypertensive and anti-atherosclerotic effects. In this narrative review, we presented the cardioprotective properties of two different approaches that go beyond anticoagulation: NOACs and NK. By combining evidence from basic research with clinical findings, we aim to elucidate the comparative cardioprotective efficacy of these interventions and highlight their respective roles in modern cardiovascular care.

## 1. Introduction

In the field of cardiovascular (CV) health, the quest for effective cardioprotective interventions continues to drive basic and clinical research. Among numerous strategies, both pharmaceutical and natural remedies have gained significant attention due to their potential to reduce CV risk factors and improve overall health. Over the past decade, the use of non-vitamin K antagonist oral anticoagulants (NOACs), also known as direct oral anticoagulants (DOACs), has brought significant progress in the management of CV diseases (CVDs). Following extensive clinical trials, NOACs have been shown to be clinically superior to vitamin K antagonists (VKAs), such as warfarin, particularly in the prevention and treatment of thromboembolic events [1]. According to the European Society of Cardiology (ESC) guidelines, NOACs are preferred over VKAs for the prevention of thromboembolism in patients with atrial fibrillation (AF) and for the treatment of venous thromboembolism (VTE) [2,3]. In addition, the use of NOACs has numerous advantages over VKAs, including fixed dosing, lack of laboratory monitoring, and fewer food and drug-to-drug interactions [1]. Currently available NOACs include the factor (F) Xa (FXa) inhibitors rivaroxaban, apixaban, and edoxaban and the FIIa (thrombin) inhibitor dabigatran. However, which of these agents is the most suitable for patients with AF remains unclear [4]. In addition, previously conducted investigations have shown differences in the incidence of atherothrombotic, ischemic events between NOACs [4]. Interestingly, randomized clinical trials (RCTs) showed an increased rate of myocardial infarction (MI) with dabigatran [5,6,7], while the FXa inhibitors rivaroxaban and apixaban reduced the incidence of MI compared to warfarin [4], which warrants further investigations.

Nattokinase (NK) is a serine protease obtained by purification and extraction from natto, a traditional Japanese soybean fermented by *Bacillus subtilis* [8]. The first research into its anticoagulant properties dates back to the 1980s and is attributed to *Hiroyuki Sumi*, who observed that natto has the ability to dissolve artificial fibrin, which was a crucial discovery in the field [9]. Following this observation, an enzyme, later identified as NK, was isolated from natto. Various experimental and clinical studies reported the positive effects of NK on the circulatory system regarding its thrombolytic and anticoagulant actions [10,11]. This enzyme showed not only fibrinolytic activity due to its ability to degrade fibrin, but also affinity as a substrate for plasmin [12]. Recent studies have shown that NK has additional cardioprotective effects, such as antihypertensive and anti-atherosclerotic effects [12]. 

**Figure 1:**
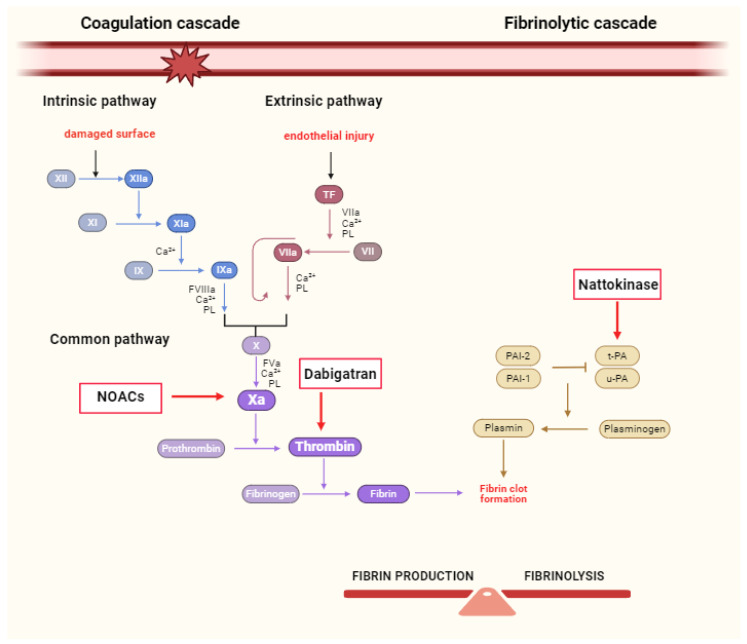
Coagulation cascade and signaling pathways of NOACs and NK. Abbreviations: NOACs—non-vitamin K antagonist oral anticoagulants; TF—tissue factor; PL—platelet phospholipids; u-PA—urokinase-plasminogen activator; t-PA—tissue-plasminogen activator; PAI-1—Plasminogen activator inhibitor-1; PAI-2—Plasminogen activator inhibitor-2.

Both NOACs and NK have anticoagulant properties affecting coagulation cascade (Figure 1), but differ largely in their origins, mechanisms of action, and clinical use. NOACs are well-known synthetic drugs deeply implemented in the therapeutic strategy for specific prothrombotic conditions. On the other hand, NK is a food-derived, natural enzyme that has potential health benefits, but is less monitored by regulatory authorities. In this narrative review, we presented the cardioprotective properties of two different approaches that go beyond anticoagulation itself: NOACs and NK. By combining evidence from basic research with clinical findings, we aim to elucidate the comparative cardioprotective efficacy of these interventions and highlight their respective roles in modern cardiovascular care.

## 2. The Role of NOACs in Coagulation Cascade

Decades of health research have paved the way to our understanding of hemostasis and thrombosis. The cascade model of coagulation, which emerged in the 1960s with the advent of biochemistry, described a sequential enzymatic process for clot formation [13]. Later, the cell-based model, integrated cellular elements into the dynamics of clot formation. These conceptual models have led to revolutions in clinical therapy, improving both procoagulant and anticoagulant treatment strategies [13].

The coagulation cascade is a tightly regulated pathway of enzymatic reactions that is essential for hemostasis and involves interactions between various plasma proteins, cellular components and membranes that ultimately lead to the formation of a stable blood clot. The initiation of the coagulation cascade is crucial in the formation of a fibrin-rich thrombus, which is necessary for effective hemostasis to stop bleeding after vascular injury [14]. The cascade can be roughly divided into two main pathways: the intrinsic pathway, which is triggered by damage to the endothelium, and the extrinsic pathway, which is triggered by tissue injury. These pathways converge upon activation of FX, which catalyzes the conversion of prothrombin into thrombin, a key enzyme in clot formation. Thrombin then cleaves fibrinogen into fibrin monomers, which polymerize and form a mesh that stabilizes the blood clot [15]. At the same time, thrombin enhances the coagulation process by activating platelets and various cofactors, which leads to the recruitment of further coagulation factors. Both FX (FXa) and thrombin are involved in common pathway of coagulation cascade [16]. The coagulation cascade is strictly regulated by anticoagulant mechanisms to prevent excessive clot formation and thrombosis. These include natural anticoagulants such as antithrombin, protein C, and tissue factor (TF) pathway inhibitor, as well as fibrinolytic mechanisms that degrade fibrin and dissolve clots once hemostasis is achieved [17]. Plasminogen activator inhibitor 1 (PAI-1), the primary inhibitor of tissue plasminogen activator (tPA), regulates fibrinolytic activity within the fibrinolytic cascade [18]. Nevertheless, the aberrant accumulation of fibrin, either qualitatively or quantitatively, in sites devoid of hemorrhage can precipitate thrombotic events [19].

### 2.1. Inhibition of FXa as a Therapeutic Strategy

Blood coagulation FX plays a central role in the coagulation cascade as a key factor in thrombin generation [20]. It is activated to FXa via the extrinsic or intrinsic pathway [21], while in the common pathway it interacts with the cofactor FVa in the presence of calcium ions to form prothrombinase, the physiological activator of prothrombin [22]. Therefore, FXa is an attractive target for pharmacological inhibition as it leads to the interruption of both the extrinsic and intrinsic coagulation pathways, as is the case with rivaroxaban, apixaban and edoxaban [23].

Rivaroxaban is an FXa inhibitor characterized by high oral bioavailability, rapid onset of action with maximum plasma concentrations within 1.5 to 2 h and minimal interactions with food and other drugs [24]. Two thirds of the active substance is eliminated by the liver (mainly via CYP3A4 and CYP2J2) and the rest is excreted via the kidneys. The onset of plasma elimination occurs with a terminal half-life of 5–9 h in young people, but 12–13 h in people older than 75 years [25]. Apixaban is another potent, highly selective and reversible FXa inhibitor that is active against both free FXa and FXa bound in the prothrombinase complex [26]. After oral administration, its bioavailability is over 50% and maximum plasma concentrations are reached within 3 to 4 h, while its half-life is approximately 12 h [27]. The main route of excretion is fecal, with a quarter of the drug excreted via the kidneys [28]. Edoxaban is also a potent, highly selective FXa inhibitor that has a high affinity for free FXa and FXa bound to the prothrombinase complex [29]. Edoxaban has a rapid onset of action and high oral bioavailability with maximum plasma concentrations after 1–2 h after oral administration and a half-life of approximately 10–14 h [30]. Most of the drug is excreted in the feces (60%) and the rest (40%) via the kidneys [31].

### 2.2. Thrombin Inhibition as a Therapeutic Strategy

FIIa, namely thrombin, has a central function in the initiation of thrombus formation, but is also involved in the activation of FV, FVIII, and FXI, which contribute to the enhancement of thrombin production, as well as FXIII, a protein involved in fibrin cross-linking and clot stabilization [32]. The primary role of thrombin is to convert soluble fibrinogen into insoluble fibrin while facilitating platelet activation [32]. According to ECS guidelines, the first oral thrombin inhibitor was ximelagatran and it was initially recommended for the prevention of VTE in major elective orthopedic surgery. However, this agent was withdrawn from the market 20 months later due to its hepatotoxicity occurring after 35 days [33]. Dabigatran etexilate is an orally active double prodrug that is rapidly metabolized to dabigatran, a potent and reversible direct thrombin inhibitor [32]. Dabigatran is a low-molecular weight molecule that binds to an active site of thrombin via ionic interactions and inhibits both free and thrombin bound to clot [32]. After oral administration, the maximum plasma concentrations are reached within 1.5–2 h [34]. In vitro studies have shown that dabigatran does not inhibit cytochrome P450 enzymes, so that drug-drug interactions are negligible [34]. Dabigatran is mainly excreted via the kidneys, while a small amount of the drug is excreted via the bile after the formation of acyl glucuronides [32].

## 3. NOACs through the Scope of Large Clinical Trials

At the time of marketing approval, none of the NOACs were novel; rather, all of the components were synthesized approximately two decades prior to their first use in humans [35]. The initial clinical evaluation of NOACs primarily involved short-term trials for perioperative prophylaxis of deep vein thrombosis (DVT) and pulmonary embolism (PE). Subsequently, phase III clinical trials, characterized by longer treatment duration, were conducted to evaluate the efficacy and safety of NOACs in the treatment of acute DVT and PE as well as in secondary prophylaxis. These studies served as the basis for the comprehensive evaluation of NOACs in large-scale clinical trials with various therapeutic indications. The main objective of these studies was to determine the comparative efficacy and safety profiles of NOACs versus conventional anticoagulant therapies, particularly warfarin [35].

The RE-LY study involved patients diagnosed with non-valvular AF and at moderate to high risk of thromboembolic stroke, randomized to receive either warfarin or one of two doses of dabigatran. This study found that both doses of dabigatran were non-inferior or superior to warfarin in preventing stroke or systemic embolism, while showing similar or lower rates of major bleeding [5]. The exceptional efficacy and safety of dabigatran over warfarin was unfortunately associated with an increased risk of MI compared to warfarin, which could be attributed to warfarin’s proven efficacy in MI prevention [5]. In parallel, the ROCKET-AF study investigated the effect of rivaroxaban in patients with non-valvular AF and an increased risk of stroke [34]. In this study rivaroxaban was found to be non-inferior to warfarin in preventing stroke or systemic embolism, while having a similar risk of major bleeding [36]. The ARISTOTLE trial involved patients with non-valvular AF and at least one stroke risk factor who were randomized to receive either apixaban or warfarin [37]. The superiority of apixaban over warfarin in the prevention of stroke or systemic embolism has been shown, accompanied by a lower incidence of major bleeding and lower mortality [37]. Finally, edoxaban was investigated in the ENGAGE-AF-TIMI 48 study, which involved patients with moderate-to-high AF [38]. The high-dose edoxaban was non-inferior to warfarin in preventing stroke or systemic embolism and had a lower risk of bleeding. On the other hand, the lower dose of edoxaban showed non-inferior efficacy but was associated with less bleeding compared to warfarin [38]. These studies have demonstrated the efficacy and safety of NOACs compared to VKAs for stroke prevention in AF, leading to their widespread use in clinical practice. In addition, several other studies have investigated the use of NOACs in other conditions such as the prevention and treatment of VTE and have provided further evidence of their efficacy and safety in these conditions. In the RE-COVER study, dabigatran demonstrated comparable efficacy and safety to warfarin in patients with acute VTE, but its administration did not require laboratory monitoring [39]. In the EINSTEIN study, rivaroxaban was shown to be effective and safe in the treatment of acute DVT and the prevention of recurrences [40]. In general, the EINSTEIN clinical development program, which included four phase III clinical trials, has demonstrated the efficacy and safety of rivaroxaban for the treatment of DVT and PE and the secondary prevention of recurrent VTE in a broad patient population. The program includes four completed Phase III trials in adult patients (EINSTEIN DVT, EINSTEIN PE, EINSTEIN EXT and EINSTEIN CHOICE) and the EINSTEIN JUNIOR program for children [41]. In addition, in the HOKUSAI VTE study, edoxaban was found to be similarly effective to standard anticoagulant treatment in patients with VTE and severe PE and was associated with less bleeding [42].

Overall, there is solid evidence for the use of NOACs over conventional VKAs such as warfarin in various clinical settings. As we have already discussed, the use of NOACs offers many advantages over VKAs, including comparable or even better efficacy and a better safety profile. These factors have led to NOACs’ upgraded position in guidelines such as those of the ESC and the European Association for Cardio-Thoracic Surgery (EACTS) [43]. NOACs are also preferred because of their simplicity of use, as they can be administered in a fixed dosing regimen without the need for regular laboratory monitoring, unlike warfarin, which requires monitoring of the international normalized ratio (INR) to ensure correct dosing and to avoid complications from over- or under-anticoagulation [44]. Nevertheless, some patients require the use of VKAs over NOACs. This mainly refers to the patients with mechanical valve prostheses (wherever they are implanted) and moderate or severe rheumatic mitral valve stenosis patients. According to the results of the RE-ALIGN study, mechanical heart valves remain the absolute contraindication to NOACs’ use due to lack of efficacy in these settings [45]. Therefore, the choice between NOACs and VKAs should be individualized based on factors such as patient preferences, clinical characteristics, and the presence of specific comorbidities or conditions where VKAs may still hold an advantage [35].

In RCTs, a lower risk of major bleeding was observed with apixaban, edoxaban and low-dose dabigatran, while similar trends were observed with rivarxaban and high-dose dabigatran compared with warfarin [5,36,37,38]. Interestingly, there are no RCTs on potential differences in bleeding risk between NOACs [43]. In a recent nation-wide cohort study, rivaroxaban was associated with an increased incidence of major bleeding compared with apixaban and dabigatran; however, rates of stroke or systemic embolism were similar for all three agents [46]. Patients with life-threatening bleeding, major uncontrolled bleeding, or patients requiring urgent reversal of anticoagulation for urgent surgical procedures are appropriate candidates for the administration of a reversal agent [47]. The Food and Drug Administration (FDA) has approved two reversal agents for NOACs: idarucizumab for dabigatran and andexanet alfa for apixaban and rivaroxaban [48].

## 4. Nattokinase—A Promising Agent for CVD Treatment

The tradition of natto consumption has persisted for over two millennia in Asian cultures. Notably, its consumption was attributed to the longevity observed in the Japanese population. Recent investigations have revealed compelling associations between increase in natto intake and a reduced risk of total CVDs mortality, specifically, due to ischemic heart diseases [49]. NK, an alkaline protease consisting of 275 amino acid residues and with a molecular weight of around 28 kDa, was identified by multiple studies as the primary active ingredient of natto [12]. More than three decades ago, Sumi and colleagues reported that NK supplementation enhances fibrinolytic activity in the plasma, as evidenced by fibrinolytic parameters and tPA production [9]. NK not only directly and effectively degrades fibrin but also augments the release of tPA, thereby increasing plasmin formation [12]. In a study conducted by Fujita and colleagues, NK showed antithrombotic activity approximately four times stronger than that of plasmin in dissolving chemically induced thrombi in the common carotid artery of rats [50]. Oral administration of NK to rats with PE reduced thrombus number and plasma euglobulin lysis time (ELT) and increased tPA, suggested a promising effect on the activation of plasma fibrinolysis in vivo [51]. The antithrombotic efficacy of NK was also confirmed in a carrageenan-induced tail thrombosis model [52]. A specific purified protein layer, NKCP, which consisted mainly of NK, had similar fibrinolytic and antithrombotic effects to heparin [53]. The fibrinolytic effects of NK are due to its ability to enhance fibrinolysis by cleaving and inactivating PAI-1 into low-molecular weight fragments [54]. On the other hand, NK directly triggers fibrin dissolution in the absence of PAI-1 [55]. Furthermore, NK successfully converts endogenous prourokinase into urokinase, also known as urokinase-type plasminogen activator (uPA) [55]. NK has also been shown to block thromboxane formation, resulting in inhibition of platelet aggregation without causing bleeding [56].

In an open-label clinical trial, NK supplementation over a 2-month period resulted in a significant reduction in fibrinogen, FVII, and FVIII levels, suggesting promising CV benefits [57]. In addition to antithrombotic, anticoagulant, and antiplatelet effects, other effects of NK have been identified in recent years, including its antihypertensive, lipid-lowering, and anti-atherosclerotic effects, as well as the potential to prevent Alzheimer’s disease, treat retinal diseases, and inflammatory bowel diseases [58]. Interestingly. antiviral effects of NK have also been observed. In a study by Oba and colleagues, natto extract was shown to inhibit bovine herpesvirus 1 (BHV-1) and SARS-CoV-2 infection [59]. Moreover, the inhibition of SARS-CoV-2 infection by natto extract was achieved by the degradation of S protein by NK [60]. Given the observed health benefits of NK, this natural, safe, effective, and inexpensive dietary supplement is a promising agent for the treatment or prevention of CVDs and beyond [55].

However, NK is not FDA-approved and cannot replace treatment with prescribed anticoagulants. Nonetheless, gathering evidence on the beneficial effects of supplementation may provide better insights into the mechanisms and at least encourage further research and large RCTs with this substance.

## 5. Cardioprotection beyond Anticoagulation—Evidence from Basic and Clinical Research

When developing a pharmacologic agent, it is important to evaluate its potential additional beneficial effects beyond its primary intended use. In comprehensive review, we will discuss the main cardioprotective effects of NOACs and NK that go beyond their primary effects on coagulation cascade, offering insights into their broader physiological impacts and therapeutic potential. A summary of selected preclinical studies regarding the cardioprotective potential of NOACs and NK is presented in Table 1 and Table 2. 

### 5.1. Effects on Myocardial Structure and Function

An unexpected increase in the rate of MI with dabigatran compared to warfarin [5] led to further clinical evaluation. However, studies addressing this issue reported conflicting results. One meta-analysis of fourteen RCTs showed a higher risk of MI with dabigatran compared to warfarin [78], while another meta-analysis of observational studies reported no difference in MI rates between these agents [79]. Furthermore, early registry studies failed to demonstrate a difference in MI risk between dabigatran and rivaroxaban [80,81,82]. Most recently, a large nation-wide cohort study reported a 2-fold increased risk of MI in patients treated with dabigatran compared to those receiving rivaroxaban or apixaban [46]. These observations suggested an increased incidence of MI with dabigatran may be due to the fact that, unlike warfarin and rivaroxaban, dabigatran increases the excretion of thromboxane, a marker of platelet activation, as shown in human [83,84] and animal studies [85]. 

Protease activated receptors (PARs) are widely distributed in cardiac muscle tissue and can be activated by various proteases [86]. Both FXa and thrombin activate different cell types via PARs, with thrombin mainly involved in the activation of PAR-1, PAR-3, and PAR-4, while FXa activates PAR-2 [16]. Activation of PAR-1 and PAR-2 induces cardiomyocyte hypertrophy, while stimulation of PAR-1 leads to myofibroblast proliferation [86]. PAR-2 signaling pathway has been involved in the regulation of myocardial hypertrophy and ischemic myocardial remodeling [87], while acting synergistically with the transforming growth factor (TGF)-β1 signaling pathway in several pathologies, including renal injury [88] and pancreatic cancer [89]. Several animal studies have investigated the effect of NOACs on myocardial function in heart disease, particularly their effect on the PAR signaling pathway. Rivaroxaban improved cardiac systolic function in the MI mouse model and reduced infarct size and cardiac mass by downregulating the mRNA expression of PAR-1 and PAR-2, as well as TGF-β and tumor necrosis factor-α (TNF-α) in the infarcted area, and of atrial natriuretic peptide (ANP) and brain natriuretic peptide (BNP) in the non-infarcted area [87]. In addition, rivaroxaban reduced cardiomyocyte hypertrophy and phosphorylation of extracellular signal-regulated kinase (ERK) in the non-infarcted area [61]. Similar effects were observed in MI rats treated with rivaroxaban, demonstrating improved left ventricular (LV) remodeling [62]. This effect is achieved through reduced PAR-2 activation and the resulting increased expression of TGF-β [62]. PAR-2 signaling pathway in myocardial fibrosis is mediated in part by upregulation of TF, which in turns increases FXa, which triggers PAR-2 activation, leading to initiation of the coagulation cascade [90]. Rivaroxaban suppressed the PAR-2 inflammatory cascade in mice with ischemic cardiomyopathy, resulting in significant cardiac benefits [91]. Low-dose rivaroxaban has been also observed to have a positive impact on LV remodeling and diastolic function in mice with transverse aortic constriction [63]. Bode and colleagues explained that the beneficial effects of rivaroxaban on cardiac function are due to the influence of two parallel signaling pathways that independently contribute to cardiac injury [64]. One of these pathways involves the anticoagulant potential of rivaroxaban, which leads to the maintenance of blood flow in the cardiomyocytes in the border zone around the infarct area and a reduction in intravascular thrombus formation. The other is related to the activity of rivaroxaban, which inhibits FXa-PAR2 signaling, preventing cardiomyocytes hypertrophy and cardiac remodeling [64]. The use of NOACs should also be investigated with regard to pulmonary arterial hypertension (PAH) and right ventricular (RV) failure. In a study by Delbeck and colleagues, treatment with rivaroxaban prevented RV dysfunction and hypertrophy in monocrotaline-treated rats, which may indicate the involvement of specific coagulation factors in the progression of PAH [92]. 

In the context of PARs regulation, rivaroxaban is definitely the most studied NOAC. However, a possible downregulation of PAR-1 expression was also observed with dabigatran treatment, followed by an improvement in coronary flow reserve and myocardial function in the pressure overload mouse model [65]. In addition, dabigatran reduced infarct size, increased arterial pressure, and reduced the no-reflow phenomenon in an experimental acute MI (AMI) model [91]. Recently, Martínez-Fernández and colleagues had shown that edoxaban treatment had no significant effect on experimental remodeling after MI, but resulted in better recovery than placebo [66]. Furthermore, treatment with apixaban also had no effect on myocardial remodeling in rats with MI-induced heart failure (HF) [93]. A meta-analysis of observational studies examining the effects of NOACs on cardiac function in patients with AF undergoing percutaneous coronary intervention declared rivaroxaban the most appropriate NOAC for the prevention of MI in patients with AF with the lowest mortality among these patients [94].

There are limited data in the literature on the positive effects of NK on myocardial function and remodeling. The cardioprotective effects of NK were observed in an experimental model of isoproterenol-induced MI. NK-treated rats had significantly lower levels of cytosolic enzymes such as troponin T, creatine kinase (CK), creatine kinase-myoglobin binding (CK-MB), lactate dehydrogenase (LDH), aspartate aminotransferase (AST), and alanine aminotransferase (ALT) [71]. Elevated levels of these enzymes are closely related to leakage of blood into the circulation due to rupture of the cell membrane and increased permeability [95]. In addition, elevated troponin T levels are considered a crucial criterion for MI diagnosis and are very useful for MI prognosis as they predict LV function, infarct area size and adverse cardiac events [96]. The ability of NK to reduce the level of these enzymes confirms its cardioprotective effects, which translate into improved cell membrane integrity, reduced myocardial necrosis and better prognosis [71]. However, additional studies are required to confirm its effect in vitro and in vivo, as well as in the human population.

### 5.2. Antihypertensive Effects

In patients with hypertension, increased levels of D-dimer, fibrinogen and prothrombin fragment 1 + 2 are observed, indicating activated coagulation processes. This suggests that both an enhanced renin-angiotensin system (RAS) and an increased tendency to form blood clots may play a role in the progression of damage to organs affected by hypertension [97,98]. Lowering blood pressure in patients with AF before starting oral anticoagulants has been shown to reduce the risk of hemorrhagic stroke [99]. Preclinical data showed that rivaroxaban significantly lowers portal pressure in rat models of liver cirrhosis by improving intrahepatic vascular resistance, increasing NO bioavailability, deactivating hepatic stellate cells and reducing intrahepatic microthrombosis [100]. These observations were of great translational importance. Further RCT reported that treatment with rivaroxaban improved survival without increasing the risk of bleeding in patients with cirrhosis and moderate hepatic impairment [101]. An earlier study investigated whether rivaroxaban may be able to alleviate renal damage in hypertension. Rivaroxaban was shown to exert a protective effect on renal podocyte damage by an activated RAS through a blood pressure-independent effect, but partly by influencing the PAR-2 signaling pathway [102]. Furthermore, there are neither experimental nor clinical studies indicating antihypertensive effects of direct FXa antagonists, suggesting that further research is needed to uncover the mechanism responsible for the antihypertensive potential of NOACs. On the other hand, both the thrombin inhibitor dabigatran and warfarin have been shown to cause a dose-dependent increase in systolic blood pressure in nephrectomies rats, which may be mediated in part by reduced thrombin activity [103]. On the other hand, NOACs can preserve endothelial function and reduce the inflammation associated with atherosclerosis [104]. Endothelial dysfunction is a hallmark of hypertension and CVDs [105]. Studies are investigating whether the effects of NOACs on endothelial function lead to improved blood pressure regulation. In an in vitro model of chronic atherosclerosis using 25-hydroxycholesterol (25-OHC)-induced human umbilical vein endothelial cells (HUVECs), treatment with dabigatran improved endothelial integrity by decreasing mRNA expression of intercellular adhesion molecule 1 (ICAM1) and vascular endothelial growth factor A (VEGFA) [106].

In contrast, blood pressure-lowering effects of NK have been described in several studies. An in vitro study reports that natto suppresses the angiotensin-converting enzyme (ACE), which is responsible for the production of the strongest vasoconstrictor angiotensin II [107]. In spontaneously hypertensive rats, the administration of NK led to a significant reduction in blood pressure [72], which has also been confirmed in human studies. The first RCT investigating the effects of NK treatment on blood pressure showed a significant reduction in both systolic and diastolic blood pressure after 8 weeks of NK treatment [108]. In addition, Jensen and colleagues reported antihypertensive effects of NK treatment in patients suffering from hypertension [109]. Although the exact mechanism for the antihypertensive effect of NK is not known, some authors showed that it was independent of plasma renin activity [109], while ACE concentrations were not significantly altered in patients receiving NK [106]. Animal studies generally support the effect of NK on the ACE inhibitor [72,110], which appears to be dose-dependent [111]. 

### 5.3. Antifibrotic and Antihypertrophic Effects

Interestingly, Guo and colleagues reported increased cardiac expression of FX in the mouse model of transverse aortic constriction. FXa induces PAR-1/-2-mediated cardiomyocyte hypertrophy and PAR1-mediated cardiac fibroblast proliferation, migration, and differentiation. The authors demonstrated that cardiomyocytes and fibroblasts produce FXa under stress conditions and that a low, non-anticoagulant dose of FXa inhibitor elicited significant cardioprotection, followed by reduced cardiac fibrosis, hypertrophy, and inflammation [63]. Edoxaban also showed effects on the FXa/PAR signaling pathway. This NOAC decreased the expression of PAR2 and reduced atrial remodeling and progression of AF in a canine HF model [67]. In addition, rivaroxaban treatment inhibited fibrotic marker expression and attenuated intracellular upregulation of TGFβ1 together with its downstream suppressor of mothers against decapentaplegic 2/3 (SMAD2/3) phosphorylation effectors in Ang II-induced fibrosis [62]. Antifibrotic effects of edoxaban were also observed in an MI rat model [91]. Edoxaban successfully decreased the percentage of collagen fibers, along with downregulation of α-smooth muscle actin (α-SMA) and collagen type 1 α1 (COL1 α 1), and expression of inflammatory markers in cardiac tissue of mice with AF [112]. Similarly, in hypertensive mice, rivaroxaban reduced LV wall thickness and fibrotic heart area as well as cardiac expression of PAR-2, TNF-α, TGF-β1 and collagen type 3 α1 (COL3 α 1) genes [90]. The same results were obtained in a study demonstrating the cardioprotective effect of rivaroxaban in a mouse model of MI due to decreased TNF-α and TGF-β levels in the infracted zones. Since TGF-β is a known fibrosis factor that modulates the secretion of proinflammatory cell molecules such as TNF-α, rivaroxaban exerts both antifibrotic and anti-inflammatory effects by blocking the expression of these cytokines [61]. In addition to fibrosis, compensatory remodeling of the heart occurs in the infarct area, but also in cells from the non-infarcted zone, leading to cardiomyocyte hypertrophy of healthy cells [113]. However, treatment of the animals with rivaroxaban for two weeks improved cardiac function and infarct segments and reduced hypertrophy in response to MI. In addition, this NOACs member reduced mRNA expression of ANP and BNP as well as p-ERK in non-infected cells [61]. Based on previous observations showing an association between increased natriuretic peptide levels in cardiac overload [114] and p-ERK expression in cardiac hypertrophy [115], it can be hypothesized that rivaroxaban achieves cardioprotection through a complex mechanism involving the prevention of compensatory cardiomyocyte hypertrophy in non-infarcted cardiomyocytes. In mice with myocardial ischemia, cardiac fibrosis was associated with higher thrombin levels, whereas treatment with apixaban reduced thrombin levels and cardiac fibrosis. The authors hypothesize that the proposed mechanism is related to PAR-1/Gq/PKC signaling [68].

New evidence suggests that NOACs have antifibrotic and antihypertrophic effects, offering promising possibilities for the treatment of CVDs. By influencing signaling pathways involved in fibrosis and hypertrophy, NOACs could not only prevent the formation of blood clots, but also attenuate myocardial remodeling associated with various CVDs, ultimately improving treatment outcomes. However, further research is needed to clarify the mechanisms underlying these effects and to fully investigate their clinical implications.

Direct studies on the effects of NK in heart disease are limited, but there is increasing evidence of their influence on fibroblasts and fibrosis in other contexts. Zhang and colleagues reported that treatment of solid tumors with NK affects fibroblasts, particularly by degrading fibronectin and inhibiting fibrosis formation by cancer-associated fibroblasts (CAFs), suggesting its potential as an antifibrotic agent [116]. The potential role of NK in cardiac fibrosis and hypertrophy is an area that requires further investigation.

### 5.4. Anti-Inflammatory, Anti-Atherosclerotic Effects and Effects on Vasculature and Endothelial Function

**Figure 2:**
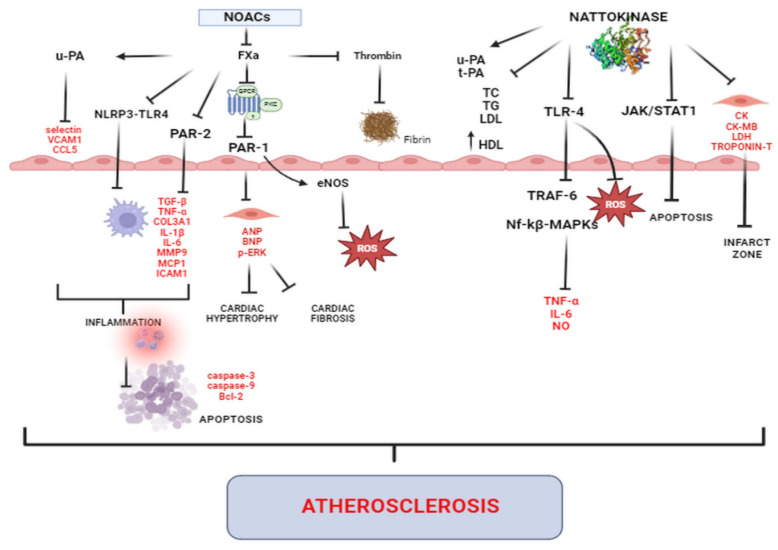
Anti-atherosclerotic mechanisms of both NOACs and NK. Abbreviations: NOACs—non-vitamin K antagonist oral anticoagulants; FXa—factor Xa; u-PA—urokinase-plasminogen activator; VCAM1—vascular cell adhesion molecule-1; CCL5—CC-chemokine ligand 5; NLRP3—NLR family pyrin domain containing 3; TLR4—toll like receptor 4; PAR—protease activated receptor; TGF-β—transforming growth factor-β; TNF-α—tumor necrosis factor α; COL3A1—collagen type III, alpha-1 chain; IL—interleukin; MMP9—matrix metalloproteinase 9; MCP1—monocyte chemoattractant protein 1; ICAM1—intercellular adhesion molecule 1; eNOS—endothelial nitric oxide synthase; ANP—atrial natriuretic peptide; BNP—brain natriuretic peptide; p-ERK—phosphorylated extracellular signal-regulated kinase; ROS—reactive oxygen species; Bcl-2—the B cell lymphoma-2; t-PA—tissue-plasminogen activator; TC—total cholesterol; TG—triglycerides; LDL—low-density lipoprotein; HDL—high-density lipoprotein; JAK—janus kinase; STAT1—signal transducers and activators of transcription 1; TRAF-6—tumor necrosis factor receptor-associated factor 6; Nf-kβ—nuclear factor kappa-light-chain-enhancer of activated B cells; MAPKs—mitogen-activated protein kinases; NO—nitrite oxide; CK—creatine kinase; CK-MB—creatine kinase-myoglobin binding; LDH—lactate dehydrogenase.

Atherosclerosis is an inflammatory arterial disease caused by elevated circulating levels of low-density lipoprotein (LDL) [117]. It is a fundamental pathological process that occurs in numerous CVDs and is known to be a major contributor to heart disease and stroke [118]. Targeted inhibition of atherosclerosis would be highly desirable for pharmacologic treatment, but represents a major challenge. Anti-atherosclerotic mechanisms of both NOACs and NK are presented in Figure 2.

Several clinical trials have reported that NOACs have a beneficial effect on cardiac damage in patients with acute coronary syndrome (ACS), as evidenced by a reduced risk of death, MI or stroke [118]. As FXa plays a decisive role in the anticoagulation cascade, it is also important in atherothrombotic diseases. In ACS, TFs activate FX and induce the conversion of prothrombin into thrombin, which is considered a strong agonist of platelet aggregation. In addition, thrombin converts soluble fibrinogen into insoluble fibrin strands, leading to thrombus formation and occlusion of the coronary artery. Consequently, inhibition of FXa can lead to the prevention of atherothrombotic events [119]. 

Although numerous studies point to the important role of the FXa-PARs signaling pathway in the proinflammatory response in various cell types and thus in the development of inflammatory diseases, there are few data on the atheroprotective effect of NOACs [120]. In a study by Hara and colleagues, rivaroxaban reduced neointima formation after vascular injury, which is closely related to the ability of this drug to inhibit macrophage activation and the resulting inflammation [121]. Administration of rivaroxaban to patients with non-ST-elevation MI resulted in a significant decrease in thrombin generation by platelets [122]. There is also evidence that rivaroxaban promotes the regression of advanced atherosclerotic plaques but also increases plaque stability, probably due to reduced activation of PARs [123]. In an experimental study on atherosclerosis conducted in mice, rivaroxaban significantly reduced plasma cholesterol levels [124]. In view of all these data, it can be assumed that rivaroxaban protects the vessels via various mechanisms, which primarily include FXa inhibition, but also the improvement in endothelial functionality, the fibrinolytic effect on endothelial cells, the reduction in instability and progression of atherothrombotic plaques, and the anti-inflammatory potential [125]. On the other hand, apixaban appears to be able to slow or stabilize the progression of atherosclerotic and calcified coronary plaques more markedly than warfarin [126]. Furthermore, apixaban was superior to rivaroxaban in attenuating coronary atherosclerosis, as evidenced by significantly less progression of calcified plaques in AF patients [127]. At the same time, edoxaban prevents maladaptive vascular remodeling in apolipoprotein E knockout (ApoE^−/−^) mice to a greater extent than warfarin [128]. The thrombin inhibitor dabigatran also has positive effects on the progression of atherosclerosis, as has been demonstrated in experimental studies [129]. It reduces pro-inflammatory M1 macrophages in atherosclerotic lesions, leading to stabilization of the atherosclerotic plaque and atheroprotective effects [130].

Investigating the anti-inflammatory potential of rivaroxaban, scientists have documented the effect of this drug on the stabilization of atherosclerotic plaques in Apo^−/−^ mice due to reduced macrophage accumulation. In addition, significantly higher expression of PAR-1 and PAR-2 was found in atherosclerotic aortas of apoE^−/−^ mice treated with rivaroxaban compared to wild-type mice, while treatment with TNF-α increased PAR-2 expression in macrophages [121]. This suggests that inflammatory microenvironments enhance the involvement of FXa-PARs signaling in macrophage activation, a central mechanism in the development of atherosclerosis. Furthermore, inflammation has been found to upregulate PAR-2 expression in endothelial cells, while FXa induces the expression of proinflammatory mediators in these cells, which in turn recruit monocytes/macrophages into the inflamed vessels, ultimately contributing to the pathogenesis of atherosclerosis [131,132]. An in vitro study was conducted to investigate how HUVECs respond to chronic inflammation induced by FXa. The results of this study showed that cell growth was triggered while their proliferation was significantly suppressed due to markedly increased proinflammatory cytokines such as IL-1β, IL-6, C-C motif chemokine ligand 2 (CCL2), and ICAM1. However, the addition of rivaraxoban to these endothelial cells reversed the effect of FXa and showed anti-inflammatory and angiogenic effects [133]. This potential of rivaroxaban was confirmed by two further studies in which anti-inflammatory properties of this agent were demonstrated due to its ability to effectively suppress inflammation by downregulating the mRNA expression of MMP9, TNFα, IL1β [121], MCP1, and ICAM1 [134]. As a molecular mechanism underlying the interplay of inflammation and blood coagulation, FXa-PARs signaling may represent a promising therapeutic approach to mitigate the inflammatory milieu in atherosclerotic lesions. Moreover, the positive anti-inflammatory effect of rivaroxaban could be supported by the activation of uPA genes [135]. Alvarez and colleagues observed increased viability of endothelial cells activated by FXa and treated with rivaroxaban. They hypothesized a strong potential of rivaroxaban to activate uPa genes that reversed the expression of FXa-induced proinflammatory genes such as selectin E, vascular cell adhesion molecule 1 (VCAM1), and CCL5 [136]. FXa inhibition by rivaroxaban has also been shown to inhibit several inflammatory signaling pathways known to be activated in HF [61]. In an experimental model of pressure overload, overexpression of inflammatory mediators was reversed with rivaroxaban, which was associated with improved atrial and ventricular remodeling and a reduction in the burden of AF [69].

Vascular calcification is an independent predictor of lifestyle risk events and mortality. The earlier IRIVASC study suggests that VKA treatment is associated with increased calcification of the coronary arteries or heart valves as seen in cardiac computed tomography (CT) scans. One of the mechanisms contributing to the reduction in intracranial hemorrhage by NOACs may be that they maintain the integrity and elasticity of the vessel walls without inducing calcification [135]. The results of the IRVASC study have shown that progression of CV calcification can be observed on CT scans regardless of VKA or NOAC treatment [137]. In retrospectively conducted observational studies, the use of rivaroxaban in newly diagnosed patients with AF was associated with less progression of valvular calcification, but also with a decrease in inflammatory markers (C-reactive protein (CRP), IL-2, IL-4, TNF-α) and fibrinolytic markers (D-dimer) [138,139,140]. The results of the post-hoc analysis of the X-VeRT study, which compared the effect of rivaroxaban and warfarin on outcomes in patients with AF, also indicate significantly lower serum levels of D-dimer and IL-6 in patients treated with rivaroxaban [141]. In patients with T2DM, rivaroxaban showed a greater improvement in endothelial function compared to aspirin, which was associated with a reduction in plasma P-selectin levels [142]. As a member of the NOACs, apixaban is usually the first choice for patients with end-stage renal disease. One study documented that exposure of endothelial cells to uremic serum resulted in increased VCAM1, ICAM1, and ROS production, leading to cell dysfunction. However, treatment with apixaban not only reduced the expression of these proinflammatory mediators, but also increased the expression of NOS3 and von Willebrand factor, which has both an anti-inflammatory and antioxidant effect [143]. These results were also documented in prospective observational human studies, which indicated a promising effect of apixaban in lowering CRP and IL-6 levels in patients with cardioembolic stroke and AF [144]. A recent RCT examined the effects of rivaroxaban and apixaban on plaque and vascular characteristics in patients with AF using cardiac CT. Plaque progression was observed with both agents, but less progression of calcified plaque and a change in positive arterial remodeling was seen in patients treated with apixaban [127]. Since epidemiologic data show that inflammation, as measured by CRP, is strongly correlated with future heart attacks and strokes, treatment with NOACs offers promising benefits in the prevention of CVDs [145]. Additional preclinical studies would be of great importance for elucidating the associated molecular mechanisms.

As a food-derived serine protease, NK is a promising anti-inflammatory agent that can prevent the formation of thrombosis. Various experimental studies investigating the anti-atherosclerotic potential of NK showed that supplementation with natto extract suppressed intimal thickening in rat femoral artery endothelial injury, which could be attributed to its thrombolytic activity [73,146]. In addition, some authors found that the beneficial effects of NK on vascular thickening were mainly due to its antioxidant and anti-apoptotic potential [147], while others showed that the anti-atherosclerotic effect of NK resulted from its direct antioxidant action in reducing lipid peroxidation and improving lipid metabolism [74]. Importantly, most experimental studies used very high doses of this supplement, so higher doses and longer duration of NK treatment would likely be required [12]. Compared with statins, NK provided better atherosclerosis management, as evidenced by a reduction in carotid artery thickness and plaque size in patients with established atherosclerotic disease [148]. Chiu and colleagues reported that NK reduces vascular inflammation by upregulating serum response factor (SRF) and thrombospondin 1 (THBS1) gene expression, leading to an increase in autophagy and a reduction in necroptosis and Nod-like receptor family pyrin domain containing 3 (NLRP3) inflammasome formation. These observations are of particular importance for the control of atherosclerosis by NK treatment [149]. However, a RCT with healthy volunteers showed that NK supplementation had no effect on the progression of subclinical atherosclerosis or on the main risk factors responsible for this process during the three-year follow-up period [150]. This unexpected discrepancy in the anti-atherosclerotic effect of NK between patients and healthy volunteers requires further investigation. The exact mechanism responsible for anti-atherosclerotic potential of NK is not known, although it could be a synergistic effect of the combination of antithrombotic, anticoagulant, antioxidant, and the lipid-lowering effects of NK or NK-rich natto extract [12]. The lipid-lowering effects of NK have also been demonstrated and probably contribute to its anti-atherosclerotic potential. In hyperholesterolemic rats, a significant reduction in total cholesterol (TC) levels was observed after 4 weeks of natto treatment [151]. In addition, NK in combination with red ginseng significantly reduced elevated serum triglyceride levels and aortic plaque area in hyperholesterolemic rabbits [152]. In patients with carotid atherosclerosis and hyperlipidemia, NK significantly lowered TC, LDL, and triglycerides (TG), but to a lesser extent than statins [148]. However, NK increased the level of high-density lipoprotein cholesterol (HDL-C), which remained unchanged during treatment with statins [148]. On the other hand, various signaling receptors are involved in the signaling pathways through which NK exerts anti-inflammatory effects. Indeed, Toll Like Receptor 4 (TLR4) expressed on macrophages appears to be a critical link between thrombosis and inflammation, as the expression of this receptor is closely associated with the release of proinflammatory cytokines [153]. Activation of the TLR4 pathway induces the translocation of nuclear factor-kappa B (NF-κB) and the transduction of mitogen-activated protein kinases (MAPKs), which stimulate innate and acquired immunity in many cells [154]. It has been found that the NF-κB/MAPK pathway can be activated by tumor necrosis factor receptor-associated factor 6 (TRAF-6), a downregulator of several receptor families with immunostimulatory properties, leading to activation of TGF-β-activated kinase 1 (TAK1) and increased release of proinflammatory mediators [155]. However, NK has been shown to inhibit the upregulation of TRAF6 and the resulting activation of the NF-κB/MAPK signaling pathway, thereby suppressing the expression of proinflammatory molecules such as TNF-α, IL-6, NO, and PAI-1, resulting in an anti-inflammatory potential [75]. As widely reported in the literature, activation of TLR4 leads to the production of reactive oxygen species (ROS) and thus to an inflammatory response. Nicotinamide adenine dinucleotide phosphate (NADPH) oxidase expressed on macrophages consists of two isoforms, of which NADPH oxidase 2 (NOX2) is most abundant on inflammatory macrophage cells. It has been documented that NOX2 is highly expressed in regions with atherosclerotic plaques [156]. Although there is little evidence for the anti-inflammatory effect of NK, one study has shown that NK has the potential to block the release of TLR- and NOX2-mediated proinflammatory mediators in macrophage cells, thereby exerting an anti-inflammatory effect [157].

### 5.5. Antioxidative Effects

Although small amounts of oxygen-derived ROS, derived from oxygen metabolism and acting as messengers, are necessary for the regulation of various cellular functions, the excessive generation of these species in combination with the depleted capacity of endogenous antioxidant enzymes leads to oxidative stress [157,158]. In a state of oxidative stress, vascular cell function is impaired by various mechanisms that affect the landscape of the coagulation cascade, endothelial cell and platelet function, and coagulation factors [159]. A previously published preclinical study suggested that oxidative stress may contribute to atherogenesis, as the atherosclerotic process was significantly enhanced in ApoE^−/−^ mice with Mn superoxide dismutase (SOD) deficiency [160]. Moreover, the discrepancy between pro- and antioxidant molecules is considered to be one of the factors in the etiology of venous thrombosis, as oxidative stress increases the expression of TFs in endothelial cells. At the same time, the most important physiological regulator of TF activity (TFPI) is blocked, which ultimately contributes to the development of the thrombotic process [161,162]. It has also been hypothesized that increased oxidation of myocardial proteins and oxidative damage to atrial tissue contribute to the development of AF, due to increased Ca^2+^ leak via the ryanodine receptor [163,164]. 

The beneficial effect of NOACs on oxidative stress has been demonstrated in numerous preclinical and clinical observational studies. For example, two in vivo studies showed a significant potential of rivaroxaban to reduce oxidative stress in an experimental model of sunitinib-induced renal injury and cardiotoxicity [165,166]. The antioxidant activity of rivaroxaban was described by a scavenging potential that led to lower malondialdehyde (MDA) levels in the animals’ tissues, followed by an increase in intracellular levels of reduced glutathione (GSH) and glutathione reductase activity. In addition, there is evidence of a significant reduction in oxidative stress in the brain of rivaroxaban-treated rats. In the experimental model of depression, rivaroxaban dose-dependently reduced the pro-oxidant molecules generated by the depressant effect, which was associated with a significant regulation of proinflammatory cytokine production [167]. Rivaroxaban also had a positive effect on redox status in other experimental models, such as liver fibrosis [99], colitis [167], testicular injury [168], and ischemia-reperfusion injury [169], due to its remarkable potential to maintain higher levels of endogenous antioxidants in experimental groups of rats. 

Using HUVECs as an experimental model, a unique preclinical study demonstrated that rivaroxaban has the potential to prevent the development of oxidative stress in endothelial cells by reducing the levels of adhesion molecules and pro-oxidant biomarkers in treated cells [170,171]. However, it has been hypothesized that this drug mediates this beneficial function via the inhibition of PAR-1, which is rapidly activated by thrombin and is closely associated with the expression of pro-oxidant molecules. Although NOACs have no direct effect on thrombin activity, the potential of rivaroxaban to reduce oxidative stress may be related to the inhibition of thrombin generation through reserved PAR-1 activation [170]. The same results were obtained in a preclinical study investigating the potential of apixaban to reduce ROS levels in HUVECs and human dermal microvascular endothelial cells (HMECs). Apixaban was able to restore the altered balance of endothelial nitric oxide synthase (eNOS) activity in this model of endothelial dysfunction, thereby restoring oxidative balance, indicating a potential protective effect on the endothelium [143]. Apart from the preclinical studies, there are only a few data from clinical studies. The positive results of the COMPASS trial on the effects of combined treatment with rivaroxaban and aspirin on CV outcomes in patients with stable coronary or peripheral artery disease [172] prompted other researchers to investigate the synergistic effects of these drugs in detail. Using an experimental model of isoproterenol-induced myocardial injury, Abedalqader and colleagues investigated the potential of dual rivaroxaban-aspirin therapy to normalize redox status in rats. In this study, rivaroxaban monotherapy improved SOD activity but also prevented lipid peroxidation by significantly reducing the level of thiobarbituric acid reactive substances (TBARS) in the cardiac tissue of rats treated with this NOAC [173]. On the other hand, co-administration of rivaroxaban and aspirin did not result in synergistic effects on the maintenance of redox balance in rats exposed to isoproterenol-induced cardiac injury [174].

As one of the mechanisms, NK exerts anti-atheroscerotic potential may be attributed to its antioxidant properties. Using the rat model of vascular injury, group of authors proved that NK prevents atherosclerosis through a direct antioxidant effect resulting in reduced lipid peroxidation and inhibition of LDL oxidation, thus improving lipid metabolism [74]. On the other hand, Chang and colleagues assigned the beneficial effect of NK on suppression of intimal thickening in animals with endothelial injury to its synergistic antioxidant and anti-apoptotic properties [147]. Indeed, the precious mechanisms by which NK prevents atherosclerosis are not fully elucidated. The present literature data proposed the collective effect of the combined anticoagulant, antithrombotic, antioxidant, and hypolipidemic actions of NK [12]. Considering that all these results were obtained from preclinical or preliminary observational studies, it is necessary to conduct future clinical trials to determine and deeply investigate whether NK affects a reduction in CV events due to its antioxidant potential. 

### 5.6. Anti-Apoptotic Effects

There is a limited evidence on the effect of NOACs on apoptosis signaling pathways. However, one study suggests that rivaroxaban may promote flap survival due to inhibition of apoptosis mediated by the TLR4/NF-κB/NLRP3 signaling pathway [175]. Namely, activation of the NLRP3 inflammasome induces TLR4, which triggers NF-κB p65 and other inflammatory immune response cells. In addition, NF-κB p65 initiates the expression of caspase-1 as well as various proinflammatory cytokines such as IL-1β and IL-18. Based on these results, rivaroxaban is thought to achieve an anti-apoptotic effect that is related to its anti-inflammatory potential [70,176]. In the rat model of myocardial ischemia, rivaroxaban markedly inhibited signaling pathways that induce apoptosis. Rivaroxaban protected the heart of rats from myocardial damage by decreasing the expression of caspase-3 and caspase-9 but increasing that of Bcl-2. This finding suggests that rivaroxaban repairs mitochondrial function through appropriate function of the Bcl-2 family of proteins, which plays an important role in preventing apoptosis by suppressing the release of cytochrome c and other proapoptotic factors from mitochondria [76,177]

A number of studies investigating NK have shown that supplementation with NK extract can lead to numerous beneficial effects, including the anti-apoptotic properties of this natural compound. NK was shown to suppress intimal thickening following vascular injury due to synergistic antioxidant and anti-apoptotic effects [147]. In addition, in an experimental model of Alzheimer’s disease, NK significantly decreased cholinesterase activity, p53, TGF-β, and IL-6 levels in association with a significantly increased Bcl-2 concentration compared to the untreated group, suggesting a neuroprotective effect due to anti-inflammatory and anti-apoptotic properties [178]. In addition, Ji and colleagues demonstrated the neuroprotective effects of NK in ischemic stroke associated with its anti-apoptotic action through activation of the JAK1/STAT1 signaling pathway. Indeed, NK exhibits anti-apoptotic potential due to the high expression of JAK1, which prevents the cell from apoptosis, and the reduced expression of STAT1, which directly activates cell apoptosis [77]. However, further investigations are warranted in relation to the anti-apoptotic effects of NK in the setting of CVDs.

## 6. Concluding Remarks and Future Directions

Clinical studies comparing NOACs with NK are currently unavailable. While NOACs have been extensively studied in large RCTs and have an established efficacy and safety profile, research on NK is still in its early stages and the evidence for its use in clinical practice is less robust. In summary, both NOACs and NK have been studied for their cardioprotective properties, but NOACs have a more extensive body of evidence supporting their efficacy and safety in clinical practice. NK shows promise as cardioprotective compound based on basic research evidence, but further well-designed clinical trials are needed to fully evaluate its efficacy and safety profile compared to standard anticoagulant therapy. If this is the case, NK could become an important agent that can be used as monotherapy or as a supplement in various CVDs.

## Figures and Tables

**Table 1:** Summary of selected preclinical studies with cardioprotective potential of NOACs.

Animal Model	Drug(s)/Dose(s)/Duration	Proposed Molecular Mechanism(s)	Cardioprotective Effect(s)	Ref.
MI mouse model	rivaroxaban 138.5 ± 50.3 mg/kg/day or vehicle 2 weeks	↓ mRNA expression of inflammatory markers (TGF-β and TNF-α in IA; ANP and BNP in non-IA) ↓ mRNA expression of PAR-1 and PAR-2 in IA ↓ the density of Iba1-positive macrophages in non-IA ↓ cardiomyocyte hypertrophy and p-ERK phosphorylation in non-IA	⮚ improved cardiac remodeling and function ⮚ ↓ LVDs and ↑ FS and IVSTd at day 7 and 14 after MI ⮚ ↓ infarct size ⮚ ↓ cardiac mass	[61]
MI rat model	rivaroxaban 3 mg/kg/day or PAR-2 10 μg/kg/day 4 weeks	↓ PAR-2 activation ↑ mRNA expression of TGF-β ↓ mRNA expressions of Col I, Col III, and α-SMA	⮚ ↓ ischemic area and improved cardyomiocite structure ⮚ improved LV remodeling and hemodynamics ⮚ ↑ LVESP and ↑ dP/dt (±dP/dT), EF, and FS ⮚ ↓ cardiac fibrosis	[62]
TAC mouse model	rivaroxaban 1 mg/kg/day or vehicle 3 weeks	↓ cardiac expression of inflammatory pathways and markers (STAT3, nF-kB, IL-1β, IL-6, and INF-γ) ↓ mRNA expression of ANP and BNP ↑ SERCA2 activity ↓ HW/TL ↓ phosphorylation of Erk1/2 and Erk5 ↓ cardiac expression of fibrotic markers (TGF-β, Col III, CTGF, MMP-2, and MMP-9)	⮚ improved LV remodeling ⮚ improved diastolic dysfunction ⮚ ↓ cardiac inflammation ⮚ ↓ cardiac hypertrophy and fibrosis	[63]
MI-induced HF mouse model	rivaroxaban 80 mg/kg/day or placebo 4 weeks	anticoagulant activity inhibiting FXa activation of PAR-2 further research is warranted	⮚ improved cardiac remodeling ⮚ ↑ EF and FS and ↓ LVPW and LVID ⮚ ↓ infarct size	[64]
PO mouse model	dabigatran 10 mg/gm or placebo 5 weeks	↓ expression of SM α-actin and PAR-1 ↓ cardiac collagen production by 15%	⮚ improved coronary flow reserve ⮚ improved global cardiac function ⮚ ↓ HW/BW ratio ⮚ ↓ cardiac fibrosis (interstitial fibrosis by 54% and perivascular fibrosis by 25%)	[65]
MI rat model	edoxaban 20 mg/kg/day or vehicle 4 weeks	↓ cardiac levels of VGEF, TGF-β1, and MMP-9 ↑ cardiac desmin levels	⮚ imporoved recovery ⮚ ↓ cardiac fibrosis	[66]
Congestive HF canine model	edoxaban 2 mg/kg/day or placebo 19 days	↓ mRNA expression of PAR-2 and fibronectin further research is warranted	⮚ ↓ atrial remodeling and fibrosis ⮚ ↓ AF progression	[67]
AF mouse model	rivaroxaban 0.01 mg/kg/day or edoxaban 0.03 mg/kg/day or placebo 2 weeks	↓ cardiac collagen fiber % ↓ cardiac expression of α-SMA and Col1a1 ↓ cardiac and blood expression of TNF- α, IL-1β, IL-6, and IL-10	⮚ ↓ cardiac fibrosis and inflammation ⮚ ↓ TC, TAG, and LDL and ↑ HDL	[68]
Myocardial ischemia mouse model	apixaban 30 or 60 mg/g/day or vehicle 4 weeks	↓ cardiac mRNA and protein expressions of Col1a1 and Col3a1 ↓ thrombin activity inhibition of Par-1 coupled Gq/PKC signaling.	⮚ ↓ cardiac fibrosis (dose-dependent) ⮚ ↑ LVSP and ↓ LVEDP, LVWI, and RVWI	[68]
TAC mouse model	rivaroxaban 30 mg/kg/day or placebo 2 weeks	↓ cardiac expression of MCP-1, Col-1, and Col-3 ↓ cardiac mRNA expression of TNF-α, IL-1β, and IL-6 ↑ anti-inflammatory M2 macrophage recruitment	⮚ ↓ cardiac fibrosis and inflammation	[69]
Myocardial ischemia rat model	rivaroxaban 2 mg/kg/day or placebo 28 days	↓ caspase 3, caspase 9, and APAF1 ↑ Bcl-2	⮚ ↓ cardiac cell death	[70]

Abbreviations: MI—myocardial infarction; mRNA—messenger ribonucleic acid; TGF-β—transforming growth factor-β; TNF-α—tumor necrosis factor α; IA—infarcted area; ANP—atrial natriuretic peptide; BNP—brain natriuretic peptide; PAR—protease-activated receptor; Iba1—ionized calcium-binding adapter molecule 1; p-ERK—phosphorylated extracellular signal-regulated kinase; LVDs—left ventricular systolic dimension; FS—fractional shortening; IVSTd—intraventricular septum thickness in diastole; Col1—collagen type 1; Coll3—collagen type 3; α-smooth muscle actin; LVESP—left ventricular end-systolic pressure; dP/dt—the ratio of pressure change in the ventricular cavity during the isovolemic contraction period; TAC—transverse aortic constriction; STAT3—signal transducers and activators of transcription 3; Nf-kB—nuclear factor kappa-light-chain-enhancer of activated B cells; IL-interleukin; INF-γ—interferon gamma; SERCA2—sarcoplasmic/endoplasmic reticulum Ca2+-ATPase; HW/TL—heart weight/tibial length; CTGF—connective tissue growth factor, MMP—matrix metallopeptidase; LV—left ventricle; HF—heart failure; FXa—factor Xa; LVPW—left ventricular posterior wall; LVID—left ventricular internal diameter; PO—pressure-overload; SM—smooth muscle; HW/BW—heart weight/body weight; VGEF—Vascular endothelial growth factor; AF—atrial fibrilation; TC—total cholesterol; TAG—triglycerides: LDL—low-density lipoproteins; HDL—high-density lipoproteins; Gq—G protein coupled-receptors q; PKC—protein kinase C; LVSP—left ventricular systolic pressure; LVEDP—left ventricular edn-diastolic pressure; LVWI –left ventricular weight indices; RVWI—right ventricular weight indices; MCP-1—monocyte chemoattractant protein-1; APAF1—apoptotic protease activating factor 1; Bcl-2—the B cell lymphoma-2.

**Table 2:** Summary of selected preclinical studies with cardioprotective potential of NK.

Animal Model	Drug(s)/Dose(s)/Duration	Proposed Molecular Mechanism(s)	Protective Effect(s)	Ref.
MI-induced HF mouse model	NK capsules 200 mg 1 mL/kg/day or vehicle 30 days	↑ cardiac CAT levels ↑ eNOS ↓ vascular congestion, necrosis and leucocyte infiltration of myocardial cells	⮚ ↓ cardiac damage and remodeling ⮚ ↓ cardiac mass ⮚ ↓ HW/BW ratio ⮚ ↓ LDH, TnT, CK, CK-MB, ALT, and AST ⮚ upregulation of antioxidant capacity	[71]
SHR model	NK water extract 100 mg/kg/day or captopril 15 mg/kg/day 8 weeks	↓ expressions of adhesion molecule and E-selectin ↓ repression of vascular inflammation	⮚ ↓ SBP and DBP	[72]
In vitro model	Incubation of endothelial cells with NK extract for 48 h at 37 °C or vehicle	↑ scavenging activity for 30% ↓ expressions of IL-1β, IL-6, and TNF-α	⮚ improved antioxidant capacity ⮚ anti-inflammatory effects	[73]
Hypercholesterolemic rats	water soluble NK fraction or placebo 3 weeks	↓ systemic inflammation response ↓ TC, TG, and LDL plasma level ↓ TBARS concentration ↑ Mn-SOD, Cu/Zn-SOD, CAT, and GSH-Px activity	⮚ ↓ TC, TG, and LDL plasma level ⮚ upregulation of antioxidant capacity	[74]
LPS-induced glomerular thrombosis in mice	NK 3000, 6000 or 9000 FU/kg or placebo 1 h before LPS	↓ renal expressions of IL-6 and TNF-α ↓ serum MDA and ↑ GSH and GSH-px levels ↓ LPS-induced NO levels in primary peritoneal macrophages ↓ NF-kβ translocation and LPS-induced NOX-2 activation ↓ TLR4-TRAF6 signaling pathway ↓ PAI-1	⮚ anti-inflammatory effects ⮚ antioxidative effects	[75]
Alzheimer’s rat model	NK 360 and 720 FU/kg or serrapeptase 10,800 and 21,600 U/kg 45 days	↓ TGF-β, IL-6, and p53 brain levels ↑ Bcl2 brain tissue level	⮚ anti-inflammatory effects ⮚ ↓ cholinesterase brain activity	[76]
MCAO rat model	NK 9.4 mg/d 4 h, 24 h or 48 h after reperfusion injury or model control group	⮚ ↓ MDA and ↑ SOD in brain tissue ⮚ ↑ cAMP level in platelets ⮚ ↑ JAK1 and ↓ STAT1 brain mRNA expression	⮚ prevent cell damage and oxidative stress ⮚ ↓ apoptosis ⮚ protection against ischemic neuronal injury	[77]

Abbreviations: MI—myocardial infarction; HF—heart failure; NK—nattokinase; CAT—catalase; eNOS—endothelial nitric oxide synthase; HW/BW—heart weight/body weight; LDH—lactate dehydrogenase; TnT—troponin T; CK—creatine kinase; CK-MB—creatine kinase-myoglobin binding; ALT—alanine aminotransferase; AST—aspartate transaminase; SHR—spontaneously hypertensive rats; SBP—systolic blood pressure; DBP—diastolic blood pressure; IL—interleukin; TNF-α—tumor necrosis factor α; TC—total cholesterol; TG—triglycerides; LDL—low-density lipoproteins; TBARS—thiobarbituric acid reactive substances; Mn-SOD—Manganese superoxide dismutase; Cu/Zn-SOD—copper/zinc-containing superoxide dismutase; GSH-Px—glutathione peroxidase; LPS—lipopolysaccharide; MDA—malondialdehyde; GSH—reduced glutathione; NO—nitrite oxide; nF-kB—nuclear factor kappa-light-chain-enhancer of activated B cells; NOX-2—NADPH oxidase 2; TLR4- toll-like receptor 4; TRAF6—tumor necrosis factor receptor associated factor 6; PAI-1—plasminogen activator inhibitor 1; TGF-β—transforming growth factor-β; p53—tumor protein P53Bcl-2—the B cell lymphoma-2; MCAO—middle cerebral artery occlusion; JAK1—janus kinase 1; cAMP—cyclic adenosine monophosphate; STAT1—signal transducers and activators of transcription 1; mRNA—messenger ribonucleic acid.
